# Case Studies in the Assessment of Microbial Fitness: Seemingly Subtle Changes Can Have Major Effects on Phenotypic Outcomes

**DOI:** 10.1007/s00239-022-10087-9

**Published:** 2023-02-08

**Authors:** Sarah B. Worthan, Robert D. P. McCarthy, Megan G. Behringer

**Affiliations:** 1 Department of Biological Sciences, Vanderbilt University, Nashville, TN USA; 2 Evolutionary Studies Initiative, Vanderbilt University, Nashville, TN USA; 3 Department of Pathology, Microbiology, and Immunology, Vanderbilt University Medical Center, Nashville, TN USA

**Keywords:** Experimental Evolution, Starvation, *Escherichia coli*, pH, Growth curves, Competition

## Abstract

Following the completion of an adaptive evolution experiment, fitness evaluations are routinely conducted to assess the magnitude of adaptation. In doing so, proper consideration should be given when determining the appropriate methods as trade-offs may exist between accuracy and throughput. Here, we present three instances in which small changes in the framework or execution of fitness evaluations significantly impacted the outcomes. The first case illustrates that discrepancies in fitness conclusions can arise depending on the approach to evaluating fitness, the culture vessel used, and the sampling method. The second case reveals that variations in environmental conditions can occur associated with culture vessel material. Specifically, these subtle changes can greatly affect microbial physiology leading to changes in the culture pH and distorting fitness measurements. Finally, the last case reports that heterogeneity in CFU formation time can result in inaccurate fitness conclusions. Based on each case, considerations and recommendations are presented for future adaptive evolution experiments.

**Supplementary Information:**

The online version contains supplementary material available at 10.1007/s00239-022-10087-9.

## Introduction

In the decades following the publication of Darwin’s theory of natural selection, evolutionary studies relied heavily on using a comparative approach. A paradigm shift in the field occurred with the advent of an experimental evolution approach, allowing for more direct observation of evolutionary changes (Morgan 1910). Within the last 30 years, utilizing microbes in experimental evolution has become increasingly popular due to their tractability and the capacity to experimentally test broad evolutionary concepts (Elena and Lenski 2003; Lenski 2017; McDonald 2019). When combined with recent advances and increased affordability of next-generation sequencing technologies, these types of experiments have become even more convenient. As such, their use has markedly increased throughout the field of microbiology, especially in the context of biomedical and engineering applications where the approach is more commonly referred to as adaptive laboratory evolution (ALE) (Dragosits and Mattanovich 2013; Lenski 2017; Sandberg et al. 2019; Zheng et al. 2021; Konstantindis et al. 2021; Mavrommati et al. 2022; Wu et al. 2022; Fait et al. 2022).

Foundational to experimental evolution is the concept of relative fitness. Following ALE experiments, researchers often want to measure the rate and extent of adaptation in evolved populations (Ayala 1965; Orr 2009). In microbes, evolved fitness is typically evaluated in two main ways: by using growth parameters derived from microbial growth curves; and head-to-head pairwise competition assays. The first method uses data obtained from growth curves as an indirect measure of fitness and is one of the most commonly used methods outside of the experimental evolution field (Warringer et al. 2003; Kugelberg et al. 2005; Paulander et al. 2009; Łapińska et al. 2022). In this approach, growth parameters, such as the maximum growth rate (V_max_), carrying capacity (K), and area under the curve (AUC), are calculated from absorbance values and used as proxies for fitness (Kugelberg et al. 2005; Hansen et al. 2007; Hegreness et al. 2008; Paulander et al. 2009; McDonald et al. 2012; Ketola and Saarinen 2015; Kang et al. 2019; Santiago et al. 2020; Frey et al. 2021). Populations with increased V_max_, K, or AUC values are considered to be more fit relative to those with lower values. Alternatively, in pairwise competition assays, fitness is directly assessed by measuring the change in the relative frequency of ancestral and evolved cells during co-culture (Lenski et al. 1991; Wiser and Lenski 2015; Lampe et al. 2019; Borin et al. 2021).

Although measuring evolved fitness appears relatively straightforward, here we present three case studies illustrating how seemingly minor variations in the experimental setup of fitness evaluations can drastically alter outcomes. These case studies are a product of phenomena observed in our lab while conducting experiments that were motivated by an interest in understanding how spatial structure and resource availability influence the evolution of microbial behavior and physiology. To this end, we commonly perform ALE experiments in a variety of culture vessels to mimic variations in spatial structure, as well as differing intervals of resource repletion resembling fluctuations of feast and famine commonly observed in nature. During a previous ALE experiment, we conditioned *E. coli* populations to three feast/famine cycles by subculturing populations into 16 × 100 mm glass culture tubes containing fresh LB broth either every day, every 10-days, or every 100-days (Behringer et al. 2020; Wei et al. 2022). We found that our results from evaluating the fitness of evolved clones and engineered mutants varied based on: (1) if fitness was evaluated directly or indirectly; (2) the type of growth vessel used for culturing strains during fitness assays and the sampling method employed; and (3) the timing at which fitness was evaluated from CFU counts. These data illustrate the importance of determining how to set up an experimental evolution protocol and highlight considerations for replicating these conditions when assessing evolved phenotypes, including the need to provide adequate pre-screening of fitness outcomes.

## Results & Discussion

### Case 1: The Effect of Vessel Type and Sampling Methods on Growth and Fitness

Fitness is routinely evaluated following an ALE experiment to characterize the magnitude of adaptation for evolved genotypes or to determine the impact of a mutation introduced into a “clean” ancestral genetic background. Due to the relatively high-throughput and inexpensive nature of estimating culture density via absorbance in a spectrophotometer, one of the most common approaches for assessing fitness is indirectly through a microbial growth curve. However, this approach comes with a long list of caveats, including the following assumptions: (1) the relationship between absorbance and CFU/ml is unchanged after evolution (i.e., no evolved changes in cell size/shape); (2) the environment in which the growth curve is measured is analogous to the evolved environment; and (3) the growth parameters used as indirect measures of fitness directly translate to fitness (i.e., there are no evolved adversarial interactions between the lines) (Concepción-Acevedo et al. 2015; Ram et al. 2019). These assumptions are often erroneous as large-scale phenotyping can result in contradictory observations, such as the relationship between absorbance and CFU/ml not being affected by evolution (Lenski and Travisano 1994; Grant et al. 2021; Marshall et al. 2022; Smith et al. 2022). Thus, for our first case, we compared three approaches for indirectly estimating fitness through a microbial growth curve. Here, we assessed three engineered mutant strains which contained either the single mutation (M1 or M2) or a double mutant containing both the M1 and M2 mutations (M1/2). These mutations affect global transcriptional regulators and had rapidly fixed in *E. coli* populations that were evolving to 100-day feast/famine cycles in an upright, shaking culture tube containing 10 ml of LB broth. We first used the V_max,_ K, and AUC values as proxies for fitness and then compared these values to fitness measurements estimated from competitive co-culture assays to gauge the consistency between methods.

**Fig. 1:**
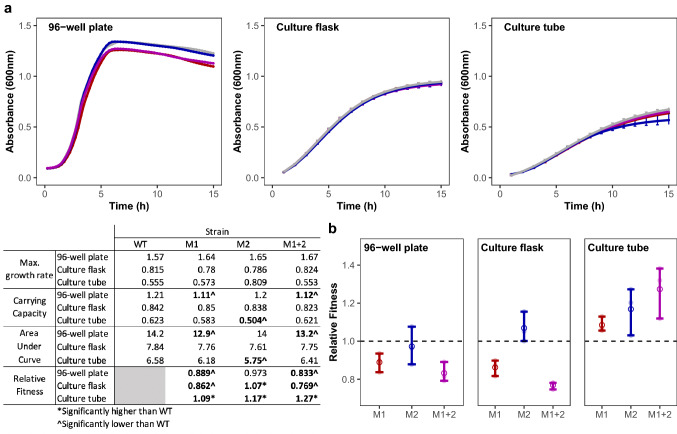
Growth patterns and fitness of engineered mutant strains and their WT ancestor vary according to growth vessel type. **a** The growth of WT (grey) and three engineered mutant strains, M1 (red), M2 (blue), M1/2 (purple), were evaluated in three types of growth vessels: a 96-well plate, culture tubes, and culture flasks over 15 h. Growth patterns of tested strains differ between growth vessels. **b** Relative fitness of M1, M2, and M1/2 in pairwise competition assays against WT ancestor after 24 h of growth in a 96-well plate, culture tube, or culture flask demonstrate variations in fitness based on growth vessel. Dashed line represents a 1:1 ratio of mutant strain and WT ancestor. Inlaid table contains maximum growth rate, carrying capacity, and area under the curve (calculated from growth curves in panel **a**), as well as relative fitness calculations (calculated from data in panel **b**) of each strain. Data points and error bars represent the mean and 95% confidence interval, respectively. All data are representative of at least three biological replicates (Color figure online)

To assess fitness outcomes calculated from growth curves, we selected three commonly used laboratory environments for microbial culture: a flat bottom 96-well plate, a 50 ml glass Erlenmeyer culture flask, and a 16 × 100 mm glass culture tube. We recorded the absorbance of cultures grown in LB broth and incubated shaking at 37 °C using a plate reader (96-well plates and culture flasks) or a spectrophotometer (culture tubes) over the course of 15 h. Since the culture environment of a 96-well plate or culture flask is well-mixed and generally homogeneous, these culture vessels were re-sampled for each time point in the growth curve. Conversely, because the spatial structure in culture tubes may represent different ecological niches that would be disrupted by vortexing, we sampled independent tube replicates for each time point. Using this setup, we measured the growth of three engineered mutants (M1, M2, M1/2) of *Escherichia coli* K-12 str. MG1655 alongside their common WT ancestor (Fig. 1a). From these data, we determined the V_max_, K, and AUC values for all four strains under each growth condition (Fig. 1a; Inlaid Table). When comparing the growth patterns of these strains using V_max_ as a proxy for fitness, none of the engineered mutants exhibited fitness values different from the WT. However, for both K and AUC, engineered mutants M1 and M1/2 exhibited significantly lower values than the WT when grown in a 96-well plate (pairwise *t*-test; M1: *P*_*K*_ = 8.56 × 10^–3^; *P*_*AUC*_ = 0.019; M1/2: *P*_*K*_ = 8.66 × 10^–3^; *P*_*AUC*_ = 0.041) and M2 exhibited significantly lower values than the WT when grown in a culture tube (pairwise *t*-test; M2: *P*_*K*_ = 0.030; *P*_*AUC*_ = 0.012). As such, with these values used as indirect fitness measures and depending on the culture vessel used, one could conclude that M1 and M1/2 have lower relative fitness than the WT strain in 96-well plates and that M2 has a slightly lower relative fitness in culture tubes.

We then compared the fitness values derived from growth analyses to an alternative method used to determine fitness, pairwise competition assays. This approach involves co-culturing two strains in known initial proportions and tracking the growth of each strain over time, where the fittest strain should increase in proportion. The two competing strains can be differentiated from each other without any effects on fitness using the *araBAD* operon as a marker (Levin et al. 1977; Lenski 1988; Lenski et al. 1991). Here, one strain in the competition contains an engineered deletion of the *araBAD* operon, whereas in the other strain the *araBAD* operon would remain intact. Then, when plated on TA agar, the strain with an intact *araBAD* operon produces a beige, or dusty pink, colony while the *ΔaraBAD* strain produces a dark red colony. To assess the accuracy of using absorbance-derived fitness estimations, we set up head-to-head competitions conducted in either a 96-well plate, culture flasks, or culture tubes between each individual strain (M1, M2, M1/2) and their WT ancestor. We then calculated their relative fitness after 24 h (Fig. 1b; Inlaid Table). In all culture vessels, inconsistencies arose between fitness outcomes measured by head-to-head competitions and fitness outcomes inferred from growth parameters. When competitions were performed in a 96-well plate both M1 and M1/2 were determined to be less fit than their WT ancestor (*t*-test, 96-well pate: *P*_*M1*_ = 0.001, *P*_*M1/2*_ = 0.001), which is consistent with fitness inferences from K and AUC, but not V_max._ Alternatively in culture flasks, head-to-head competitions reveal M1 and M1/2 to be significantly less fit than the WT strain, and M2 to be significantly more fit (*t*-test, culture flasks: *P*_*M1*_ = 0.028; *P*_*M2*_ = 0.01; *P*_*M1/2*_ = 2.45 × 10^–3^), despite none of the engineered mutants exhibiting growth patterns that suggested differences in fitness from the WT. Finally, all engineered mutants exhibited greater fitness than the WT strain when competed in culture tubes (*t*-test, *P*_*M1*_ = 0.006; *P*_*M2*_ = 0.014; *P*_*M1/2*_ = 0.007), in contrast to the growth curves where K and AUC values predicted reduced fitness in strain M2.

**Fig. 2:**
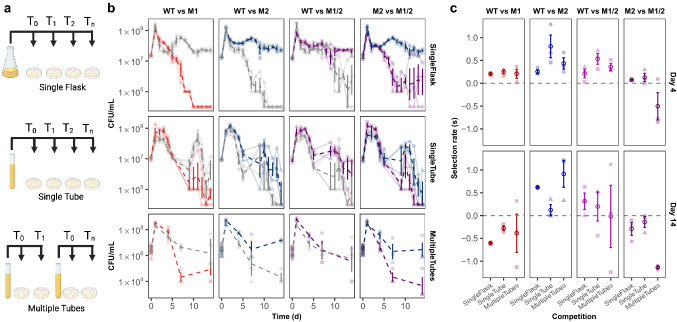
Competition dynamics between three engineered mutants and their WT ancestor differs based on sampling method. **a** Three sampling methods used for competitions included repeatedly sampling a single competition grown in a flask (Single Flask) or tube (Single Tube) or sampling a competition tube once in which one competition tube is sampled per time point (Multiple Tubes). **b** Viable plate counts, expressed as CFU/ml, of competitions between engineered mutants M1 (red), M2 (blue), and M1/2 (purple) and their WT ancestor (grey) using the three different sampling methods (single flask, single tube, multiple tubes) over a period of 14 days reveal variations in competition dynamics. Emboldened dashed lines represent the average CFU/ml of the lightly shaded dashed lines of the three replicates (circle, triangle, square) measured over 14 days of continuous growth. **c** Selection rate (*s*) for each matchup on day 4 and 14 of the pairwise competitions when performed using three different sampling methods. Lightly shaded data points represent each biological replicate (circle, triangle, square) while the emboldened open circle represents the mean. All error bars represent 95% confidence intervals (Color figure online)

As our mutations of interest initially evolved in the culture tube environment, we expected that the engineered mutant strains would outcompete the ancestral WT strain in culture tubes. Comparing these results to the competitions conducted in culture flasks and 96-well plates suggests that these mutations provide a specific benefit that may be related to the spatially-structured ecology in the culture tube environment. We previously found that cultures maintained in our glass culture tubes experience environmental heterogeneity that leads to the development of subpopulations adapted to spatial and nutritional niches (Behringer et al. 2018). As such, we hypothesized that inhibition or disruption of the heterogeneous culture environment would alter the fitness outcomes of the competitions. Moreover, the effects of disrupting the heterogeneous culture environment could compound over time and produce confounding results in experiments designed to assess fitness over time. Because our lab is interested in how microbes evolve in resource-limited conditions, we routinely evaluate the competitive fitness of our mutants over 14 days without resource replenishment to capture the competition dynamics into long-term stationary phase. Although our results clearly illustrate that large-scale differences in fitness exist between culture flasks and tubes for these engineered mutants, it may be tempting for researchers to continuously resample from culture tubes when assessing fitness in starvation conditions. Especially as setting up individual time point competitions can feel tedious, while resampling from a single culture tube could be perceived as a way to increase throughput, reduce materials, and preserve incubator space. Therefore, we evaluated fitness measurements from pairwise competition assays performed with slight modifications in the sampling method. Specifically, our competition experiments between mutant strains and their WT ancestors were performed either by resampling from a single 50 ml Erlenmeyer culture flask (Fig. 2a, Single Flask), resampling from a single glass culture tube (Fig. 2a, Single Tube), or sampling from multiple tubes for individual time point competitions (Fig. 2a, Multiple Tubes). We then extended our previous competitions that were conducted in either single flasks or single tubes to be sampled repeatedly over 14 days. Additionally, the lower-throughput multiple tube competitions were sampled on days 1, 4, 7, and 14. At each time point, the CFU/ml of each strain was determined through viable plate counts (Fig. 2b). Here, measurements taken from resampling single tubes reveal that both competitors perform poorly in these conditions, as total CFU/ml (or the sum of both competitors’ CFU/ml) is drastically decreased compared to the other sampling methods by the end of the 14-day period (ANOVA with Tukey’s HSD, *P*_*single flask v. single tube*_ < 1.39 × 10^–11^; *P*_*single flask v. multiple tubes*_ = 0.07) (Fig. S1). We expect this poor performance is likely due to the variability in the environmental conditions caused by the periodic disruption of the tube’s spatial structure, as opposed to the constant disruption or constant maintenance of spatial structure associated with single flasks or multiple tubes, respectively.

To score the competitions, we calculated the selection rate on days 4 and 14 of the competition (Fig. 2c), instead of relative fitness values which were calculated for day 1 (Fig. 1b). As cultures have entered death phase and long-term stationary phase at the 4- and 14-day time points, the selection rate is a more appropriate metric to score long-term competitions (Lenski et al. 1991). Again, since M1 and M2 represent mutations that arise very early during evolution to starvation conditions in culture tubes, we expected that these engineered mutant strains would outcompete the WT ancestor when cultured in the multiple tube environment as this environment is the most analogous to the evolved environment. However, we had no prior expectation for how the performance of the engineered mutants would be affected based on the sampling method. The most striking effects of sampling method are observed in competitions between M2 vs. M1/2. Here, the double mutant M1/2 is less competitive than M2 in the multiple tubes sampling method than the other methods at day 4, but ultimately this difference was not significant (*t*-test, *P* = 0.160). But, by day 14 the differences in selection rate were more pronounced and significantly lower in multiple tubes than in single flasks (*t*-test, *P* = 0.023) and single tubes (*P* = 0.011). Other effects of sampling method can be observed in competitions between WT and M2. Although not statistically significant, M2 exhibits the highest mean selection rate in the single tube sampling method on day 4. But on day 14, the single tube sampling method resulted in the lowest mean selection rate for M2 and the multiple tube sampling method, which should be closest to the environment in which M2 evolved, had the highest mean selection rate. These results, combined with the results from growth curve assays, illustrate the importance of fully considering how one should assess fitness following an evolution experiment as the identity of the growth vessel or sampling method can impact growth parameters and affect interpretations of competitive fitness.

### Case 2: The Effect of Vessel Material on Microbial Physiology and Fitness

**Fig. 3:**
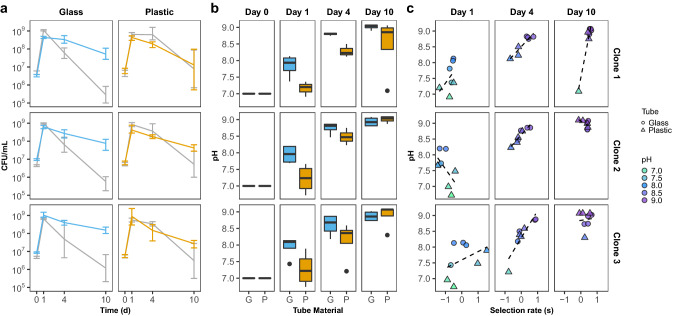
Culture vessel material affects competition dynamics, culture pH, and fitness of evaluated clones Three individual evolved clones were co-cultured and competed against their WT ancestor strain in either glass or plastic culture tubes for a period of 10 days. **a** Growth (as measured by viable plate counts expressed as CFU/ml) of co-cultures containing the WT ancestor (grey) and 3 selected individual evolved clones (Clone 1, Clone 2, Clone 3) grown in either glass (blue) or plastic (orange) culture tubes over 10 days show differences in competition dynamics. Data points represent mean with error bars showing 95% confidence interval. **b** Box plots with quantile distribution of culture pH measurements on 0, 1, 4, and 10 days of competition when co-cultures were grown in either glass (G, blue) or plastic (P, orange) culture tubes. Co-cultures performed in plastic tubes are more acidic than those in glass tubes on days 1 and 4 of competition (Wilcox test; *P*_*Day1*_ = 0.002; *P*_*Day4*_ = 0.006; *P*_*Day10*_ = 0.68). **c** Plots comparing the selection rate (s) of evolved clones and their culture pH when grown in glass (circle) or plastic (triangle) culture tubes after 1, 4, and 10 days of competition. Dashed line represents the best fit line. On day 4 of competition, there is a significant positive correlation between selection rate and culture pH. All averages are based on at least three biological replicates (Color figure online)

Our previous case illustrates the effect of the growth vessel on fitness values derived from growth analyses and pairwise competition assays. While it may be obvious that microbes can exhibit different physiological behaviors when grown in a culture flask versus a culture tube, it is less obvious how physiology can be affected between culture tubes constructed of different materials. Recently, in an effort to streamline our pairwise competition assays and conform to current biosafety recommendations (CDC 2021), we investigated the effect of substituting plastic polypropylene culture tubes in place of our typical borosilicate glass culture tubes when evaluating fitness. Although this appears to be a minuscule change of materials, we were surprised to find considerable differences in the competition dynamics between competition assays performed under starvation conditions in either glass or plastic culture tubes. Here, three individual evolved clones were competed against their ancestor in either glass or plastic culture tubes for a period of 10 days (Fig. 3a). These clones were originally isolated from an experimental population that was cultured in glass tubes while evolving to 10-day feast/famine cycles (Behringer et al. 2022). When grown in their native environment of glass tubes, the evolved clones exhibit reduced individual fitness on day 1, but convincingly outcompete the WT ancestor on days 4 and 10. Conversely, when cultured in plastic tubes, the evolved clones appear less fit than the WT ancestor on both days 1 and 4 and exhibit only modest increases in selection rate over the WT ancestor on day 10.

We hypothesized that one possible reason for these substantially different fitness outcomes could be due to the impact of the pH of the culture media throughout the 10-day competition assay. It is well documented that as cultures grown in LB broth progress through the growth phases, they also experience shifts in pH associated with the metabolites used as energy sources (Sezonov et al. 2007; Sánchez-Clemente et al. 2020). The major sources of energy present in LB broth are amino acids which when metabolized lead to the production of ammonia and alkalinization of the culture media, typically within 24 h. However, due to the inclusion of yeast extract, there are also trace amounts of fermentable sugars in LB broth, which can result in a slight acidification of the media during early exponential growth (Zarkan et al. 2019; Sánchez-Clemente et al. 2020). Given this dynamic pH environment and the potential that adaptation to resource limitation in LB broth could be associated with increased tolerance of alkaline environments, we were concurrently interested in the pH of the cultures throughout the competition assay. We found, as a whole, culture media from competitions performed in plastic culture tubes were significantly more acidic, compared to the competitions performed in glass tubes, after 1 and 4 days of competitive incubation, but not after 10 days of competition (Wilcox test; *P*_*Day1*_ = 0.002; *P*_*Day4*_ = 0.006; *P*_*Day10*_ = 0.68; Fig. 3b). Interestingly, the differences in culture pH could also be correlated to selection rate (Fig. 3c). On day 4 of the competition assay, there was a strong positive correlation between the measured culture pH and clone selection rate (Pearson’s *R*_*Clone 1*_ = 0.928, *P* = 0.0025; *R*_*Clone 2*_ = 0.932, *P* = 0.00074; *R*_*Clone 3*_ = 0.898, *P* = 0.0024), suggesting that clones performed better in more alkaline conditions.

It is still unclear as to why growing cultures in glass versus plastic culture vessels have such large effects on the pH of the media. One possibility could be that the dimensions of the plastic tubes could differ from the glass tubes and result in differences in culture aeration (Juergensmeyer et al. 2007). An investigation into the physical structure of the tubes provides some insight. Although the inner dimensions of both types of culture tubes are the same, the outer diameter of the glass culture tubes measures 15.74 ± 0.03 mm in diameter, while the plastic culture tubes are slightly wider measuring 16.36 ± 0.05 mm. When the tubes are placed in a standard test tube rack during incubation and shaken upright at 180 rpm, the glass tubes tend to rattle as they fit less snugly within the test tube rack. Since the plastic tubes have a slightly wider outer diameter than the glass tubes, they have less space to shake within the rack and thus remain in more of a static position compared to the glass tubes. The difference in shaking patterns between glass and plastic tubes suggests that cultures grown in the plastic tubes would be subject to less aeration compared to cultures grown in the glass tubes and could lead to alterations in growth patterns as previously reported (Juergensmeyer et al. 2007).

**Fig. 4:**
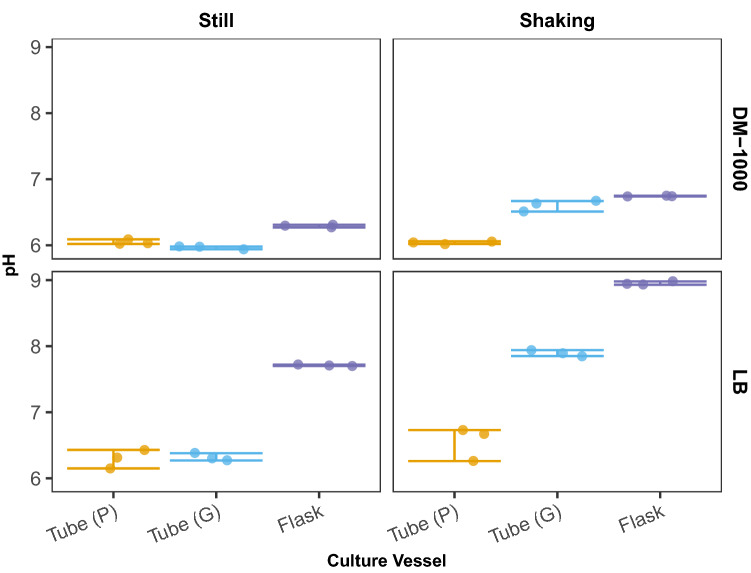
Measurements of media pH when cultured in various conditions. The media pH of WT cultures grown in either LB or DM-1000 broth in tubes made of either plastic (P, orange) or glass (G, cyan), as well as flasks (purple) was measured following 24 h of growth in either still or shaking conditions. In both medias, cultures grown in plastic tubes are acidic in both still and shaking conditions, while cultures grown in glass tubes are more acidic when incubated still than when incubated in shaking conditions. Data points represent three biological replicates and error bars reflect the 95% confidence interval (Color figure online)

To gain insight into how aeration of culture vessels affects pH, and to determine if these observations extend to other media types, we inoculated three culture vessels (plastic tubes, glass tubes, and flasks) containing 10 ml of either LB or DM-1000 broth (Davis-Mingoli broth with 0.1% glucose). All culture vessels were incubated at 37 °C under two aeration conditions (still or shaking upright at 180 rpm). Plastic tubes were checked to ensure their caps were secured in the aerobic culture position. After 24 h of incubation, we measured the pH of the culture media and observed that significant differences existed among the culture vessels. Moreover, these differences varied depending on whether cultures were incubated in still or shaking conditions (Fig. 4). Under still conditions in both media types, plastic and glass tubes exhibited similar, slightly acidic pH values (DM-1000: ANOVA with Tukey’s HSD; *P*_*plastic* v. glass_ = 0.22); LB: ANOVA with Tukey’s HSD; *P*_*plastic* v. glass_ = 0.99) and culture in flasks produced slightly alkaline conditions (DM-1000: *P*_*flasks vs. plastic*_ = 8.38 × 10^–5^; *P*_*flasks vs. glass*_ = 4.61 × 10^–6^; LB: *P*_*flasks vs. plastic*_ = 9.75 × 10^–8^; *P*_*flasks vs. glass*_ = 1.15 × 10^–7^). Alternatively in shaking conditions, glass tubes and flasks both increased in alkalinity (DM-1000: *P*_*plastic* v. glass_ = 1.07 × 10^–8^; *P*_*flasks vs. plastic*_ = 8.74 × 10^–10^; LB:*P*_*plastic* v. glass_ = 1.79 × 10^–7^; *P*_*flasks vs. plastic*_ = 1.86 × 10^–10^) compared to plastic tubes which again produced a slightly acidic pH that resembled what was observed in still conditions (DM-1000: *P*_*still vs. shaking*_ = 0.99; LB: *P*_*still vs. shaking*_ = 0.184). As a final test to confirm that the reduced rattling of plastic culture tubes during shaking incubation is a major contributor to the acidification of the media, we added a foam insert to the base of the tube rack to eliminate rattling in both glass and plastic tubes. Here, when glass tubes were incubated under shaking conditions with the addition of a foam insert, the pH of the cultures was found to be more acidic than when incubated shaking without the foam insert (Fig. S2a) (DM-1000: *P *_*glass*_ = 8.14 × 10^–7^; LB: *P *_*glass*_ = 3.67 × 10^–8^). However, the pH of these cultures did not reach the extent of acidity observed when glass tubes were incubated in still conditions. This indicates that there are additional factors independent of aeration leading to a reduction in culture pH and we can’t fully discount an effect of culture vessel material.

Under certain conditions, such as reduced aeration, *E. coli* can produce significant amounts of acetate and other mixed acids during exponential growth as a byproduct of fermentation (Wolfe 2005; Li et al. 2014; Basan et al. 2015; Millard et al. 2021). As such, when cultured in a less aerated vessel, cells would be exposed to less oxygen which could lead to acidification. Thus, in addition to measuring the culture pH, we also determined acetate concentrations of the culture media and observed an expected general trend of acetate concentrations negatively correlating with the culture pH (Fig. S2b). For both LB and DM-1000, there were no significant differences in acetate concentrations between shaking or still conditions when cultures are grown in plastic tubes (shaking vs. still, DM-1000: *P*_*plastic*_ = 0.99; LB: *P *_*plastic*_ = 0.38). However, for glass tubes, cultures only produced significantly more acetate while incubated with still conditions in DM-1000 (shaking vs. still, DM-1000: *P*_*glass*_ = 0.005), but flasks produced the least acetate while shaking with both medias (shaking vs. still, DM-1000: *P*_*flask*_ = 0.006; LB: *P*_*flask*_ = 0.0006). Because of these patterns of acidification and alkalization, researchers may desire to add buffers to culture media hoping to avoid changes in pH during culture. However, it is important to be aware that buffering is not a magic cure for these variations in environment. To illustrate the limitations of buffering media, we buffered the LB media with one of two commonly used buffers: either 50 mM of BIS–TRIS propane or 50 mM of HEPES. We then tested these buffered LB medias in our environment that experiences the most extreme alkalization: shaking culture flasks (Fig. S2c). However, even after only 24 h of growth, all cultures contained media with a pH approaching 8.0 (LB- BIS–TRIS propane: 7.79, LB- HEPES: 7.93), indicating that the buffering capacity of BIS–TRIS propane and HEPES was not enough to resist changes in pH due to metabolic products. It is also important to note that the addition of buffers can also have unintended effects on microbial cultures as HEPES can over-induce dam methyltransferase activity in *E. coli* and Tris buffers can disrupt *E. coli* membranes (Irvin et al. 1981; Hülsmann et al. 1990). Taken together, these results suggest that factors such as culture aeration, media pH, and even culture vessels that appear interchangeable but made of different materials, can produce very different environmental conditions which in turn can affect the evaluation of fitness.

### Case 3: The Effect of CFU Enumeration Timing on Fitness Assessment Accuracy

**Fig. 5:**
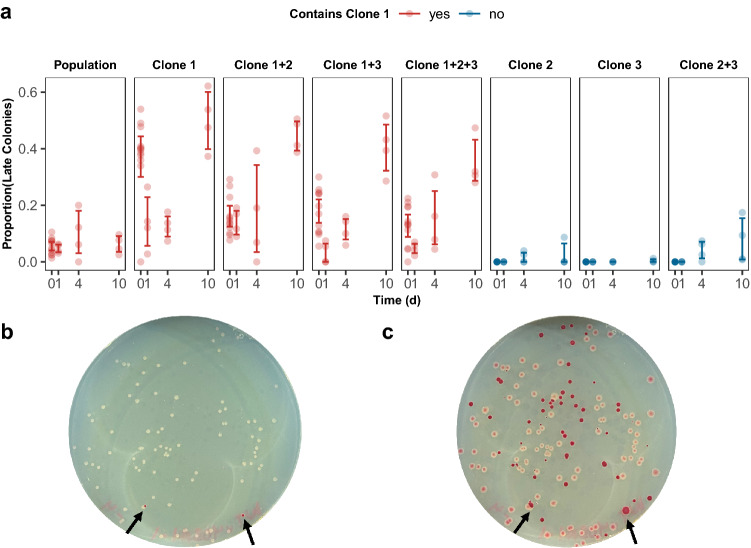
Heterogeneity of colony development in evolved lines **a**The proportion of colonies appearing after 24 h of incubation (late colonies) in various shown monocultures or co-culture competitions against their WT ancestor that either contain Clone 1 (red) or do not contain Clone 1 (blue) after 0,1,4, and 10 days of growth. Competitions which included Clone 1 displayed an increase in the proportion of late colonies. Each data point represents the proportion of colonies appearing on TA media after 24 h of incubation prepared from serially diluted competition tubes. Error bars represent 95% CI. Representative plates after **b** 24 h and **c** 48 h of incubation with arrows denoting red colonies (Color figure online)

The final case presented here regarding considerations of fitness evaluations involves the timing at which CFUs are recorded throughout a competition assay. Traditionally in competition assays, CFUs are enumerated following a 24-h incubation at 37 °C (Lenski et al. 1991; Lampe et al. 2019; Borin et al. 2021). However, while performing competition assays on clones isolated from an experimental population evolved to 10-day feast/famine cycles (Behringer et al. 2022), we observed considerable heterogeneity in the development of colonies during incubation. Here, it was not uncommon to observe “late blooming” colonies that would appear absent on the plate after 24 h, but visible by 48 h. To assess the degree of heterogeneity, competitions between this population and their ancestor were performed and the number of CFUs was tallied after 24 and 48 h of incubation (Fig. 5). When a revived sample of the population was competed against the ancestor, the majority of colonies were present within a 24-h incubation and between 10 and 20% of colonies appeared after this incubation time across a 10-day competition.

To pinpoint the source of this heterogeneity, we obtained three discernable clones within this population (Clones 1, 2, & 3), performed competitions between various combinations of these three clones and their ancestor, and tallied CFUs following 24 and 48 h of incubation (Fig. 5). Here, we determined that co-cultures containing Clone 1 resulted in the largest percentage of colonies appearing at 48 h of incubation, implying that Clone 1 is responsible for these “late blooming” colonies. (Fig. 5, red). In some competitions with Clone 1, up to 60% of the colonies could be observed as “late blooming”. As such, depending on when CFUs were enumerated, “late blooming” colonies could result in a significant underestimation of the selection rate, as large as 0.35/day. Prior research has linked “late blooming” colonies to long lag times, which could be due to the presence of persisters (Vulin et al. 2018) or as a response to stress (Leimer et al. 2016). Overall, these data illustrate two main cautionary points. First, depending on the experimental setup of the competition assay, it is worth considering increasing the incubation time for tallying CFU to eliminate the possibility of overlooking late colonies. Second, when working with evolved populations, it is critical to evaluate both the fitness of the population as a whole and the fitness of isolated clones. Examining fitness both ways can reveal interesting phenotypes, and in the case of heterogeneity, if that phenotypic heterogeneity is stochastic or genetically encoded (van Boxtel et al. 2017). Lastly, while it may be easy to dismiss this heterogeneity as a phenotype specific to evolution in starvation conditions, we have also seen similar heterogeneity in daily transferred cultures that were evolved in 50 ml flasks (data not shown). As such, it is good practice to screen competition plates for “late-blooming” colonies regardless of the evolutionary conditions.

## Conclusions

Here we discussed three cases illustrating that variations in how fitness is assessed following an ALE experiment can significantly affect conclusions about evolutionary outcomes. Our first case illustrates a few key points regarding practical considerations for measuring and defining fitness. In many cases, V_max_, K, and AUC values derived from absorbance measurements during growth analyses are used as indirect measures of fitness, but using this method as an indicator of fitness comes with a few caveats. The greatest of which is that growth-related parameters assume that microbial growth in isolation is an accurate predictor of competitive fitness. However, in many cases, fitness is more complex and a manifestation of multiple factors which would not be captured by evaluating growth parameters alone. Yet, measuring growth rate or carrying capacity can still be appropriate for evaluating phenotypes, particularly for industrial applications where an increased growth rate or yield (carrying capacity) may be the desired outcome. In these contexts, our data also indicate differences in growth yield depending on the culture vessel used. This suggests that different genotypes may exhibit differing growth behaviors due to more subtle factors, such as aeration. (Fig. 1).

Beyond industry, performing growth analyses using a 96-well plate format has increased in popularity in academic contexts, due to its ease and increased throughput (Hall et al. 2014; Stevenson et al. 2016; Kurokawa and Ying 2017; Krishnamurthi et al. 2021). Yet, caution should be exercised when justifying this decision as growth in a 96-well plate environment may not accurately reflect the evolved environment, nor may the use of this indirect method accurately predict competitive fitness as others have also noted discrepancies between fitness estimates derived from growth curves and competition assays (Concepción-Acevedo et al. 2015). When using a more direct method for fitness evaluations, such as a pairwise competition assay, performing this assay using conditions that best align with the evolved environment is crucial. Deviating from the evolved environment can greatly affect relative fitness, specifically in the type of the growth vessel used for the competition, as well as disruption of the spatially heterogeneous cultures when grown in culture tubes (Fig. 2). Moreover, although not addressed in this study, there are experimental conditions that promote diversification and the evolution of cooperative behaviors (Behringer et al., 2022). In these cases, the performance of isolated clones can depend on the presence of their complementary cooperators, and their fitness can vary greatly in isolation than in the context of within their evolving population. Thus, while Case 1 illustrates the importance of considering how abiotic factors can impact the evaluation of fitness, biotic factors can also have significant effects.

Our second case represents another seemingly subtle change, a switch from glass to plastic culture tubes, which leads to large differences in fitness values derived from pairwise competitions. Regarding biosafety, there has been a push toward utilizing single-use culture vessels and/or materials. The current recommended standard of microbiological practices is to substitute glassware for plasticware when feasible for all biosafety levels (CDC 2021). We show that the switch from our typical glass culture tube to a seemingly identical plastic culture tube with slightly different physical dimensions alters the pH and acetate concentrations of the media, due in part to factors such as aeration differences between culture tubes, thereby affecting selection rate and fitness conclusions (Fig. 3; Fig. 4). Although our investigation into this case suggests that the physical dimensions of the plastic tubes’ outer diameter plays a role in the observed changes in the media pH, we cannot discount that additional factors related to the plastic material may also be at play. Thus, while the switch to plastic materials may be feasible and inconsequential in some experiments, we recommend at a minimum to consider any potential physiological effects, such as changes in culture pH, associated with a change from glass to plastic culture vessels.

Finally, the third case presented here showcases the effects of incubation time on relative fitness calculations in pairwise competition assays. It is important to consider phenotypic factors such as colony formation time, as inadequately addressing this factor can lead to inaccurate fitness outcomes if CFUs are tallied prematurely (Fig. 5). Thus, we recommend proper screening of evolved strains for the emergence of subpopulations, or any phenotypic heterogeneity, to rule out the possibility of “late bloomers”. Furthermore, if colony heterogeneity is present, we suggest amending experimental procedures, such as extending incubation time. This will help to alleviate any discrepancies due to colony formation, allowing for a more accurate enumeration of CFUs and subsequent fitness outcomes.

In conclusion, these three case studies illustrate that fitness should ideally be assessed in the exact evolved condition. However, that doesn’t mean that the phenotypes observed in different culture vessels are invalid. Instead, data collected from culturing in different conditions reveal unique insights into microbial behavior and physiology. For instance, culturing mutants in plastic culture tubes in place of glass revealed pH adaptation as a possible mechanism of adaptation that may not have otherwise been investigated. Furthermore, a greater understanding of what traits are being selected for in the evolved environment, such as pH adaptations, can help to generate hypotheses regarding how specific mutations contribute to fitness and reveal questions that warrant further exploration.

## Materials and Methods

### Bacterial Strains and Populations

All bacterial strains used were derived from PFM2, a prototrophic derivative of *Escherichia coli* str. K-12 MG1655 (Lee et al. 2012). For Case 1, we created engineered mutant strains to isolate mutations that fixed during experimental evolution to repeated cycles of 100-day feast/famine. Strain M1 was created by cloning a Rho R109H substitution into a PFM2 Δ*araBAD* background via the Church protocol (Mosberg et al. 2012), while strain M2 and M1/2 were generated by moving ydcI783(del)::kan from the Keio collection strain JW5226 (Baba et al. 2006) with P1 vir transduction onto a PFM2 background (M2) or the M1 background (M1/2) (Saragliadis et al. 2018).

For cases two and three, all clones and populations were derived from Population 403 after 300 days of evolution to 10-day feast/famine cycles (Behringer et al. 2022). Briefly, we grew 10 ml cultures in 16 × 100 mm culture tubes and incubated them at 37 °C shaking upright at 180 rpm. Every 10 days, the cultures were diluted 1:10 by transferring 1 ml of the culture to 9 ml of fresh LB-Miller broth (10 g/l tryptone, 5 g/l yeast extract, 10 g/l NaCl). Prior to each passage, we vortexed the cultures to ensure adequate transfer of population. Every 100 days, we froze a 1 ml aliquot of the population in 40% glycerol and stored them at -80 °C until further analysis. At the 300-day time point, we isolated clones to assist with the phasing of SNPs from metagenomic sequencing data. This was accomplished by streaking out the evolved population on LB agar, selecting eight random colonies and then re-streaking these colonies again on LB agar to confirm isolation. Clones were genotyped and assigned to distinct ecotypes by next-generation sequencing. Clone 1 is 403–1, Clone 2 is 403–2, and Clone 3 is 403–5. Isolated clones were frozen in 40% glycerol and stored at -80 °C until needed.

### Growth Analyses

To examine how vessel type affects growth parameters, we assessed growth in three vessels: 96-well plates (Falcon^®^ REF 353072), 16 × 100 mm glass culture tubes (VWR No. 47729–576), and 50 ml glass Erlenmeyer culture flasks (PYREX^®^ No. 4980). Prior to growth analysis, we selected individual colonies of each strain and inoculated them into 10 ml of LB broth in 16 × 100 mm glass tubes for overnight incubation at 37 °C and shaking upright at 180 rpm (Thermo Scientific MAXQ 6000).

For 96-well plates, we used a Synergy H1 Plate Reader (Biotek) to analyze growth. A volume of 150 µl of LB broth was inoculated with 1.5 µl of overnight culture in each well so that every strain had three biological replicates. The 96-well plate underwent a 15-h program at 37 °C with continuous orbital shaking at 807 cpm. Initial and final absorbance (600 nm) readings were taken, as well as intermittent absorbance readings every 15 min. For glass flasks, the Synergy H1 Plate Reader was used to assess growth every hour by measuring the absorbance (600 nm) of 100 µl aliquots transferred from the growth flasks to a 96-well plate. Culture flasks contained 10 ml of LB broth inoculated with 100 µl of overnight culture and were incubated at 37 °C and 180 rpm for 15 h. A total of three biological replicates for each strain was used. For glass tubes, we assessed growth by measuring the absorbance (600 nm) of 1 ml aliquots that were transferred from the growth tubes to a plastic cuvette using a benchtop spectrophotometer (VWR V-1200). Glass tubes contained 10 ml of LB broth inoculated with 100 µl of overnight culture and were incubated at 37 °C and 180 rpm shaking until the required time point absorbance reading. To preserve any potential effect of ecology on *E. coli* growth dynamics, we prepared individual culture tubes for each strain for every time point and vortexed them at intermediate speed for 10 s prior to aliquoting to the cuvette. We then measured the absorbance for a total of 15 h for three biological replicates of each strain. Growth curve parameters and statistics were calculated with the R package Growthcurver (v.0.3.1) which fits growth data to a logistic model (Sprouffske and Wagner 2016).

### Pairwise Competition Assays

To calculate relative fitness, we performed pairwise competition assays as previously described (Levin et al. 1977; Lenski 1988; Lenski et al. 1991). Here, head-to-head competitions between two strains were evaluated in cultures grown in the same medium for up to 14 days. During this time, the proportion of both strains within the competition was monitored by removing an aliquot of the co-culture, serially diluting in PBS buffer, and performing viable plate counts on TA agar (10 g/l tryptone, 1 g/l yeast extract, 5 g/l NaCl, 16 g/l agar, 10 g/l L-arabinose, 0.005% tetrazolium chloride). In order to distinguish between strains, it was ensured that one competitor contained an intact *araBAD* operon, while the second competitor had a Δ*araBAD* genotype. When plated on TA agar, strains with a Δ*araBAD* genotype produce dark red colonies and strains with a functional *araBAD* operon appear as light pink colonies.

For Case 1, we inoculated overnight cultures of WT and mutant strains from colonies picked from freshly streaked LB agar plates obtained via glycerol stocks. Competitions were performed in three types of vessels: flasks, culture tubes, and a 96-well plate. For competitions in flasks and tubes, we used three sampling methods: single flask, single tube, and multiple tubes. For single flasks and tubes, one growth vessel was used for the entirety of the assay. For multiple tubes, individual growth vessels were inoculated for each sampling time point. Each competition was initiated by mixing 50 µl of each overnight culture in 10 ml LB broth for an approximate 50:50 starting proportion of each strain. Before each competition, we measured the absorbance (600 nm) of overnight cultures and if large discrepancies between co-culture densities were noted, we adjusted their volumes to normalize for cell density. Once both cultures were added to the growth vessel and gently vortexed for 10 s, we removed a 100 µl aliquot, serially diluted the sample in PBS buffer, and then plated them on TA agar to determine the T_0_ CFU/ml. For competitions in a 96-well plate, a 150 µl aliquot was transferred from the inoculated culture tube to the corresponding well in the plate. All growth vessels were incubated at 37 °C with 180 rpm shaking. Subsequently, during each time point, we vortexed each culture (except competitions grown in flasks and 96-well plates), removed a 100 µl aliquot, serially diluted again in PBS buffer, and determined viable plate counts using TA agar. We enumerated colonies after 24 and 48 h of incubation at 37 °C.

For Case 2, we inoculated WT and clone overnight cultures from colonies picked from freshly streaked LB agar plates obtained via glycerol stocks. Pairwise competitions including clones were performed in either glass or plastic culture tubes (Greiner #187261) using the aforementioned “multiple tube” setup. Each competition was initiated by mixing 50 µl of each overnight culture in 10 ml LB broth for an approximate 50:50 starting proportion of each strain. Before each competition, we normalized overnight cultures for cell density, when applicable, same as described in Case 1. Once both strains were added to the growth vessel and gently vortexed for 10 s, we removed a 100 µl aliquot, serially diluted them in PBS buffer, and plated them on TA agar to determine the T_0_ CFU/ml. At each time point, we vortexed cultures for 10 s, removed a 100 µl aliquot, serially diluted in PBS buffer, and determined viable plate counts using TA agar. We enumerated colonies after 24 and 48 h of incubation at 37 °C. Immediately following sampling, we measured the culture pH as described below.

For Case 3, population overnight cultures were inoculated directly from the glycerol stock to maintain a representative sample of the population. Overnight cultures for clones were inoculated as described in Case 2. We performed competitions using different combinations of Clones 1, 2, and 3, as well as the population as a whole, using the “multiple tube” format described in Case 1. Thereafter, competitions were inoculated, incubated, and sampled as described in Case 2. We enumerated colonies after 24 and 48 h of incubation at 37 °C.

For all cases, we calculated relative fitness (*W*) and selection rate (*s*) as previously described (Lenski et al. 1991). Relative fitness (*W*) was calculated by dividing the natural log of the ratio of final CFU/ml over initial CFU/ml for both strains using the following equation:

$$W = \frac{{\ln {{Strain\,E_{Final} } \mathord{\left/ {\vphantom {{Strain\,E_{Final} } {Strain\,E_{Initial} }}} \right. \kern-0pt} {Strain\,E_{Initial} }}}}{{\ln {{Strain\,A_{Final} } \mathord{\left/ {\vphantom {{Strain\,A_{Final} } {Strain\,A_{Initial} }}} \right. \kern-0pt} {Strain\,A_{Initial} }}}},$$

where Strain E is the evolved or engineered strain and Strain A is the ancestor or reference strain. A relative fitness value of 1 implies neutral fitness, while *W* > 1 implies Strain E is fitter than Strain A. Selection rate (*s*) was calculated by finding the natural log of the ratio of final CFU/ml over initial CFU/ml for both strains, finding the difference, and dividing by the day of competition using the following equation:

$$s = \frac{{\ln {{Strain\,E_{Final} } \mathord{\left/ {\vphantom {{Strain\,E_{Final} } {Strain\,E_{Initial} }}} \right. \kern-0pt} {Strain\,E_{Initial} }} - \ln {{Strain\,A_{Final} } \mathord{\left/ {\vphantom {{Strain\,A_{Final} } {Strain\,A_{Initial} }}} \right. \kern-0pt} {Strain\,A_{Initial} }}}}{day}$$

A positive selection rate implies Strain E is more fit over time than Strain A, while a selection rate of zero implies equal fitness over time.

### Measuring pH of Culture Media and Assessing Acetate Concentrations

For the competitions in Case 2 performed in either glass or plastic tubes, we recorded the pH of the media at each time point directly following sampling as described above. For Case 2, we measured the media pH of cultures in six environments: static 50 ml flasks, static glass tubes, static plastic tubes, shaken plastic tubes, shaken glass tubes, and shaken 50 ml flasks. Each growth vessel was inoculated with a WT colony previously grown on LB agar. Cultures were grown in either LB broth or DM-1000 (30.6 mM K_2_HPO_4_, 14.7 mM KH_2_PO_4_, 7.5 mM (NH_4_)_2_SO_4_, 1.7 mM Na_3_C_6_H_5_O_7_(H_2_O)_2_, 0.1% glucose (w/v)). When buffered media was used, the buffers BIS–TRIS propane or HEPES were added to LB media for a final concentration of 50 mM. The pH was then adjusted using 1 M HCl until a measurement of 7.0 was reached. Following inoculation, we incubated each growth vessel in either a shaking (180 rpm) or still (0 rpm) environment at 37 °C. All pH measurements were taken using an ORION STAR A214 pH/ISE (Thermo) meter at room temperature. For each measurement, the pH electrode was submerged into the culture and the pH measurement was recorded once a stable reading occurred. We performed weekly calibration of the pH meter to ensure accurate pH measurements. Measurements of culture tube and tube rack dimensions were taken using a caliper (Mitutoyo 500-197-30) in triplicate with variance expressed as standard error.

To determine if secretion of acetate from *E. coli* cells into the spent media was contributing to the observed changes in pH we measured acetate concentrations from spent LB broth or DM-1000 in each of the six culture environments. Briefly, we incubated bacterial cultures at 37 °C for 24 h before removing two 1 ml culture aliquots for analysis. We used the first aliquot to determine the culture density based on absorbance (600 nm) and harvested the supernatant from the second aliquot via centrifugation at 10,000 rpm for 5 min before assessing acetate concentrations with the EnzyChrom Acetate Assay Kit (BioAssay System) according to kit instructions. We then normalized acetate concentration by culture density.

### Statistical Analyses

All plots and statistical analyses were generated using R studio. Code and information about the specific packages is available on GitHub at https://github.com/BehringerLab/SubtleEffectsFitnessPaper.

## Supplementary Information

Below is the link to the electronic supplementary material.

Supplementary file1 (PDF 23 KB)

Supplementary file2 (PDF 10 KB)
