# Supplementary figures and images for: Case Studies in the Assessment of Microbial Fitness: Seemingly Subtle Changes Can Have Major Effects on Phenotypic Outcomes
Source: J Mol Evol. 2023 Feb 8;91(3):311–24. doi: 10.1007/s00239-022-10087-9 (PMC10276084; doi:10.1007/s00239-022-10087-9)

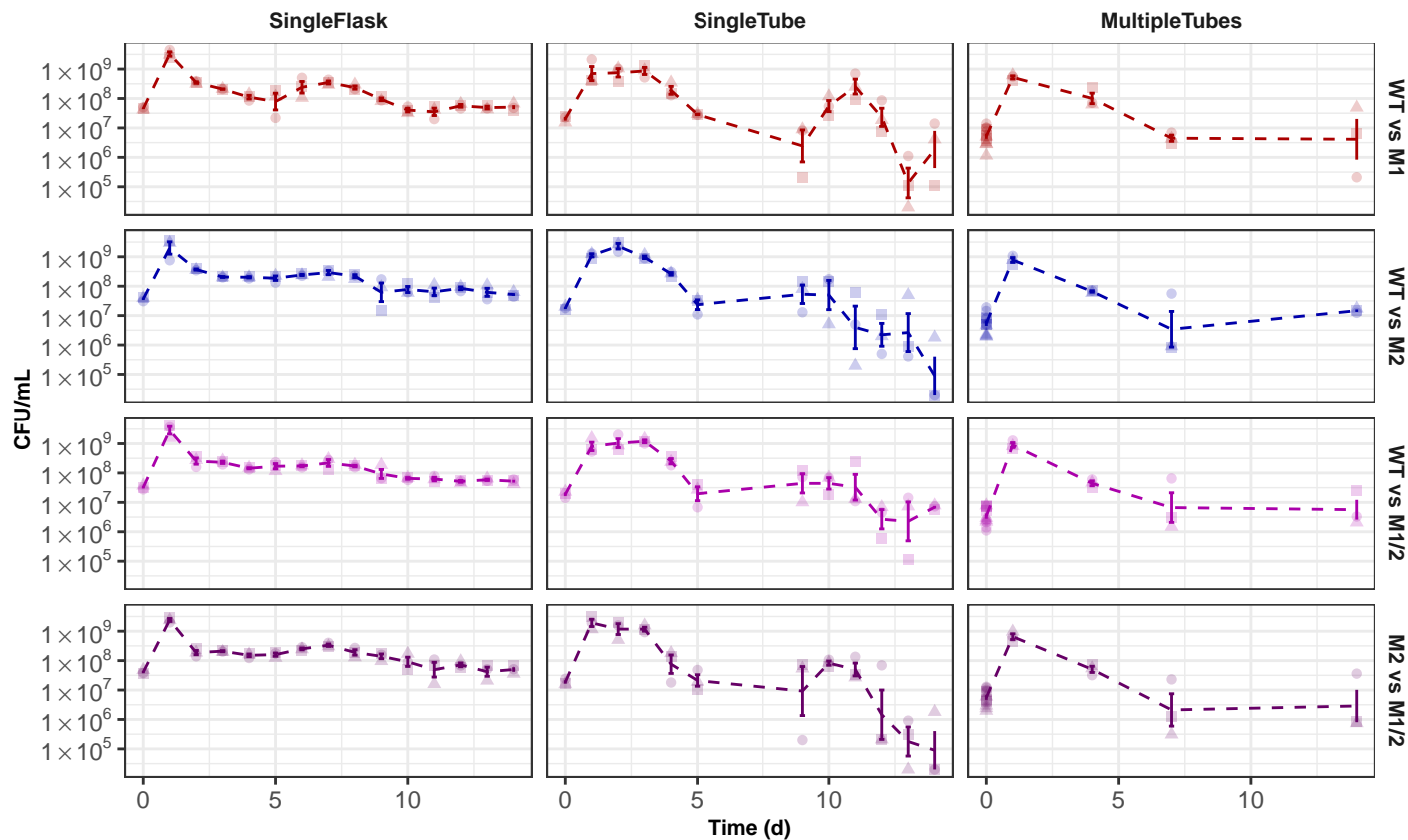

Supplement: Supplementary file 1 — Supplementary file1 (PDF 23 KB) [file 239_2022_10087_MOESM1_ESM.pdf]

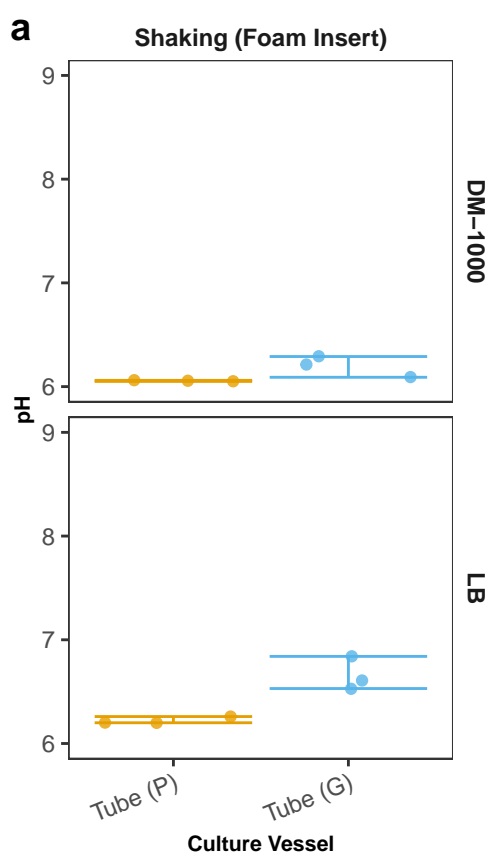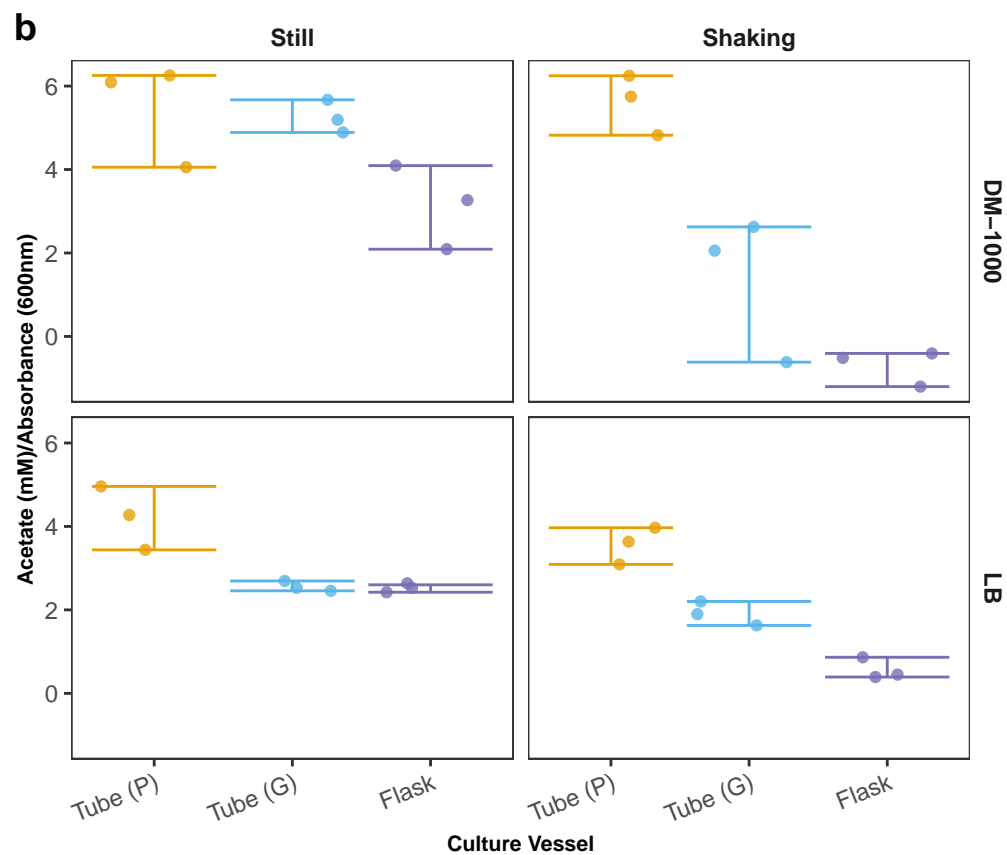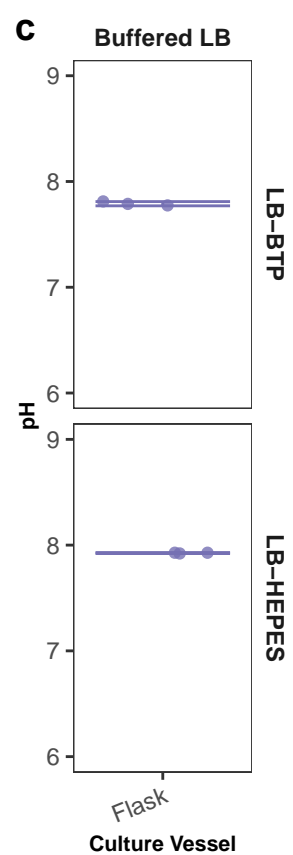

Supplement: Supplementary file 2 — Supplementary file2 (PDF 10 KB) [file 239_2022_10087_MOESM2_ESM.pdf]
